# Estimating the Cost-Effectiveness of Switching to Higher-Valency Pediatric Pneumococcal Conjugate Vaccines in the United Kingdom

**DOI:** 10.3390/vaccines11071168

**Published:** 2023-06-28

**Authors:** Michele Wilson, Aaron Lucas, Diana Mendes, Andrew Vyse, Boglarka Mikudina, Carole Czudek, Gillian Frances Ellsbury, Johnna Perdrizet

**Affiliations:** 1 RTI Health Solutions, 3040 East Cornwallis Road, P.O. Box 12194, Research Triangle Park, Morrisville, NC 27709, USA; mwilson@rti.org; 2 Pfizer Ltd., Walton Oaks, Dorking Road, Surrey, Tadworth KT20 7NS, UK; diana.mendes@pfizer.com (D.M.); andrew.vyse@pfizer.com (A.V.); boglarka.mikudina@pfizer.com (B.M.); carole.czudek@pfizer.com (C.C.); gillian.ellsbury@pfizer.com (G.F.E.); 3 Pfizer Inc., 66 Hudson Blvd E, New York, NY 10001, USA; johnna.perdrizet@pfizer.com

**Keywords:** pneumococcal disease, vaccines, cost-effectiveness, pneumococcal vaccination, economics

## Abstract

Currently, the 13-valent pneumococcal conjugate vaccine (PCV13) is administered under a 1+1 (1 primary dose) pediatric schedule in the United Kingdom (UK). Higher-valency PCVs, 15-valent PCV (PCV15), or 20-valent PCV (PCV20) might be considered to expand serotype coverage. We evaluated the cost-effectiveness of PCV20 or PCV15 using either a 2+1 (2 primary doses) or 1+1 schedule for pediatric immunization in the UK. Using a dynamic transmission model, we simulated future disease incidence and costs under PCV13 1+1, PCV20 2+1, PCV20 1+1, PCV15 2+1, and PCV15 1+1 schedules from the UK National Health Service perspective. We prospectively estimated disease cases, direct costs, quality-adjusted life-years (QALYs), and incremental cost-effectiveness ratio. Scenario analyses were performed to estimate the impact of model assumptions and parameter uncertainty. Over a five-year period, PCV20 2+1 averted the most disease cases and gained the most additional QALYs. PCV20 2+1 and 1+1 were dominant (cost-saving and more QALYs gained) compared with PCV15 (2+1 or 1+1) and PCV13 1+1. PCV20 2+1 was cost-effective (GBP 8110/QALY) compared with PCV20 1+1. PCV20 was found cost-saving compared with PCV13 1+1, and PCV20 2+1 was cost-effective compared with PCV20 1+1. Policymakers should consider the reduction in disease cases with PCV20, which may offset vaccination costs.

## 1. Introduction

Pneumococcal disease is a major clinical and economic burden in the United Kingdom (UK) [1,2]. Pneumococcal infection may be categorized as invasive (isolation of *S. pneumoniae* from blood or another normally invasive sterile location) or non-invasive [3]. Invasive pneumococcal disease (IPD) is less prevalent but more costly per case than non-invasive disease.

The first pneumococcal conjugate vaccine (PCV), 7-valent PCV (PCV7), was licensed in the UK in 2006 for pediatric vaccination. It covered the seven serotypes 4, 6B, 9V, 14, 18C, 19F, and 23F. In 2010, the UK replaced PCV7 with the 13-valent PCV (PCV13), covering PCV7 serotypes and the additional serotypes 1, 3, 5, 6A, 7F, and 19A. PCVs have led to substantial reductions in the disease burden. Indeed, before PCV7 was licensed, there were over 8000 annual cases of IPD in the UK; and after PCV13’s introduction, the UK reported roughly 4000 annual cases of IPD [4].

Historically, the UK has advised two priming doses (at two months and four months of age) and a booster dose at one year of age for both vaccines (a 2+1 schedule). After the 2+1 schedule was implemented, there was a substantial reduction in disease incidence caused by PCV-covered serotypes [5,6]. With high adherence to the 2+1 schedule and postulated equivalency between a 1+1 and 2+1 schedule, the program was modified to be a 1+1 schedule in January 2020 [7].

Since the implementation of PCV13, non-PCV–covered, serotype-specific disease incidence has increased [8]. This increase was modulated by COVID-19 disease prevention measures, though IPD incidence has largely rebounded to pre-COVID-19 levels in recent months [9,10].

Many cost-effectiveness models have been developed to assess the impact of PCVs in the UK [11,12,13]. Dynamic transmission models (DTMs) are well suited for modeling pneumococcal disease. DTMs capture both the direct and indirect effects of disease propagation, and they allow an estimate of the predictive behavior of the disease. Previous DTMs have modeled PCV13 versus PCV7 and PCV13 in different dosing schedules [7,12,14].

The 15-valent PCV (PCV15) and 20-valent PCV (PCV20) are two newly developed vaccines that are potential options for implementation in the pediatric program in the next one or two years. PCV15 covers serotypes 22F and 33F in addition to the serotypes covered in PCV13. PCV20 contains the additional serotypes in PCV15 along with 8, 10A, 11A, 12F, and 15B serotypes.

There are few studies that model the usage of PCV15 or PCV20 [15,16,17]. One study, developed for the UK, found that PCV20 would save costs and reduce disease [18]; however, that study focused on adults. To our knowledge, this cost-effectiveness analysis is the first to model higher-valency PCVs, focused on the pediatric population in the UK.

The objective of this study was to estimate the clinical and economic impact of switching to PCV20 in either a 2+1 or 1+1 schedule for pediatric vaccination in the UK. We also compared implementing PCV20 with continuing PCV13 in a 1+1 schedule or switching to PCV15.

## 2. Materials and Methods

### 2.1. Model Structure

We adapted a previously developed two-part epidemiological and economic model to analyze the impact of vaccination programs on pneumococcal disease and economic burden [11]. The first part of the model estimates IPD incidence in the population. The second part estimates the non-invasive disease incidence and associated economic burden of pneumococcal disease states derived from the IPD incidence.

**Figure 1:**
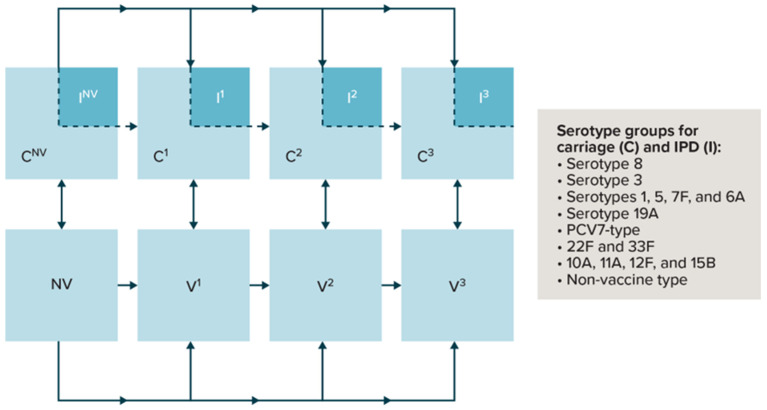
Overview of the model. IPD = invasive pneumococcal disease; NV = no vaccination; V = vaccine dose; C = carriage acquisition; I = IPD development; PCV7-type = 7-valent pneumococcal conjugate vaccine; V = vaccination. Note: Superscripted numbers indicate the vaccine dose number. Compartments illustrate a schematic look at dynamics for individuals in a given dose compartment. The population in the compartments without carriage acquisition is represented in the lower row. Compartments relevant to vaccine doses (both carriage and susceptible) are further stratified into “no immunity” and “full immunity” induced by vaccine effectiveness, age group, and serotype group.

The model to estimate IPD incidence is a deterministic DTM (Figure 1). Briefly, as described previously, individuals are born “susceptible” and may acquire a serotype and become pneumococcal carriers (C) [11]. Acquiring a serotype may happen to any susceptible individual. The model assumes carriage of only one serotype at a time and no cross-serotype protection. Carriage acquisition causes individuals to become infectious for a duration of time. After serotype acquisition, a percentage of carriers experience IPD and are treated appropriately. After being treated for IPD, individuals go back to being susceptible (an SIS [susceptible-infected-susceptible] model). Disease transmission is therefore driven by carriage prevalence. Carriers are assumed to clear the serotype after a given duration. Vaccination history is compartmentalized by no vaccination (NV), receipt of one primary dose (V^1^), receipt of two primary doses (only applicable to the 2+1 schedule) (V^2^), and receipt of a booster dose (V^3^). Individuals are split into compartments based on vaccination history, age, type of vaccine received, immunity level of the vaccine (full immunity or no immunity), and serotype group. Individuals pass through these compartments during the model run according to the dosing schedule, adherence to dosing, waning of the vaccine, carriage of a serotype, and aging.

The model considers the following age groups: <5 years; 5–17 years; 18–49 years; 50–64 years; and ≥65 years; however, the model results are split into more granular age groups for the <5 years group to capture differences in dosing schedules. The model includes four vaccines (both historical and anticipated): PCV7, PCV13, PCV15, and PCV20. To model these vaccines, serotypes are partitioned into the following groups: PCV7-type; serotype 3; serotype 19A; serotypes 1, 5, 6A, and 7F; 22F and 33F; serotype 8; serotypes 10A, 11A, 12F, and 15B; and non–vaccine-covered serotypes (i.e., non-PCV20 serotypes).

The economic model calculates non-invasive disease from the DTM and imposes costs and utilities on the health outcomes from the DTM and the calculated non-invasive disease cases. The model estimates non-invasive disease (pneumococcal pneumonia and otitis media) and deaths over time as a linear function of the IPD incidence output. Specifically, we assumed a constant proportional change in the incidence of pneumococcal community-acquired pneumonia (CAP) and otitis media incidence relative to IPD incidence. As a conservative assumption, we assumed otitis media occurs only in children under 5 years of age, and we did not include outpatient pneumococcal pneumonia in the model. Costs and utility weights are applied to cases of IPD, pneumonia, and otitis media. The model also tracks individuals vaccinated so that total vaccination costs may be estimated. The results of the model include total costs, cases of disease, life-years, and quality-adjusted life-years (QALYs) for the UK population over a 5-year time horizon. Costs and outcomes are discounted at 3.5% per year [19].

### 2.2. Model Inputs

Table 1 lists the key model input parameters obtained from published literature. Adherence to doses was based on National Health Service (NHS) data, and the contact matrix was taken from Mossong et al. [20] (see Supplementary Material). Historical IPD incidence was obtained from published data [8]. After the total number of cases of IPD is estimated, the model distributes these cases into meningitis and bacteremia using proportions from van Hoek et al. [12] and Mohanty et al. [21].

The model estimates unobservable parameters through a calibration procedure, fitting historical IPD incidence in England and Wales to IPD incidence output by age and serotype. Calibrated parameters in the model include:

- Table 2 displays vaccine effectiveness against overall IPD and against carriage. In the model, $VE_{OI}=1-(1-VE_{IPD})(1-VE_{C})$, where $VE_{OI}=$ vaccine effectiveness against IPD overall, $VE_{IPD}=$ the vaccine effectiveness against IPD given carriage, and $VE_{C}=$ the vaccine effectiveness against carriage. We assumed the vaccine effectiveness for PCV20 minus PCV13 serotypes were equivalent to the vaccine effectiveness inputs for serotypes 1, 5, 7F, and 6A. Moreover, when vaccines had in common a particular serotype group for coverage, the protective effect was equivalent across vaccines. We calibrated these vaccine effectiveness estimates and visually inspected them so that they were within the bounds of the published literature [35].
- The probability of acquiring serotype carriage given contact with a carrier, by age and serotype group, absent vaccination.
- The mean duration of protection for each dose (Table 2).
- The mean duration of carriage by age and serotype group.

Details on the calibration and the estimates for other calibrated parameters can be found in the Supplementary Material.

Parameters to estimate non-invasive disease were taken from published literature. All-cause hospitalized CAP was estimated using data from hospital episode statistics (HES) [24] and Mohanty et al. [21]. We assumed 36.6% of adult pneumonia is pneumococcal [28]. All-cause otitis media cases were estimated using data from The Health Improvement Network (THIN) [26]. The proportion of otitis media cases attributable to *S. pneumoniae* was assumed to be 20% [36,37], as the causative pathogen is neither routinely assessed nor identified [38], and 20% is often used in modeling studies after PCV13 implementation [39,40,41].

We used vaccine prices from the British National Formulary [42] and administration fees from NHS [43]. Costs of disease are in 2021 UK pounds and were obtained from publicly available databases. Costs for hospital admissions due to IPD (bacteremia and meningitis) and CAP were taken from the national schedule of NHS costs 2020/2021 [31] using the average costs and the number of finished consultant episodes with relevant Healthcare Resource Group (HRG) codes (further details are in the Supplementary Material). We conservatively excluded outpatient CAP costs from the model. Otitis media costs were estimated from NHS reference costs [31], with mild otitis media incurring an outpatient pediatric ear, nose, and throat physician and moderate/severe cases incurring an average of the outpatient and short stay costs for minor ear procedure (HRG code CA54B) and a follow-up with an outpatient ear, nose, and throat physician.

Age-specific utility estimates for the UK population were sourced from the published literature [33]. As pediatric-specific disutility weights are lacking [44], we used adult estimates for disease cases: IPD and hospitalized CAP from Mangen et al. [34] and otitis media from van Hoek et al. [12]. All-cause mortality by age was obtained from national vital statistics [23]. Case-specific (IPD and hospitalized pneumonia) mortality was estimated using the published literature [29].

The model was run to compare the current PCV13 1+1 program with a 1+1 and 2+1 program for both PCV15 and PCV20. In a 1+1 program, the first priming dose occurs at 3 months of age followed by a booster dose at 1 year. In a 2+1 program, priming doses are administered at 2 and 4 months of age.

A recently published article by Lewnard et al. [45] found that protection against carriage afforded by the booster dose in a PCV13 1+1 program may be less than that in a 2+1 program. We thus assumed that, in a 1+1 program, the vaccine effectiveness against carriage for the booster dose was reduced in PCV15 and PCV20. We applied the odds ratio of acquiring a PCV13-covered serotype in a 1+1 series versus a 2+1 series to calculate this reduction for PCV7-covered serotypes and the additional serotypes covered in PCV15 and PCV20. Specifically, we increased the odds of carriage acquisition by 2.96 for PCV7-covered serotypes and 2.05 for the additional serotypes in PCV15 and PCV20. Conservatively, we did not apply the reduction in the vaccine effectiveness against carriage for a 1+1 booster for PCV13-unique serotypes 1, 3, 5, 6A, 7F, and 19A since these serotypes had been covered with a PCV13 2+1 schedule previously.

### 2.3. Model Analyses

Base-case cost-effectiveness results are reported over a 5-year time horizon for the general population from the UK National Health Service perspective. We estimated cost-effectiveness (assuming a willingness-to-pay threshold of GBP 20,000 per QALY gained [46]) of vaccination considering the following 5 vaccine schedules: PCV13 1+1; PCV15 1+1, introduced in 2023; PCV15 2+1, introduced in 2023; PCV20 1+1, introduced in 2024; and PCV20 2+1, introduced in 2024.

We performed a one-way sensitivity analysis on the net monetary benefit (NMB) of PCV20 1+1 versus PCV20 2+1 and PCV13 1+1 versus PCV20 2+1. In addition, we completed scenario analyses (Table 3).

According to guidelines for conducting dynamic transmission modeling, we did not include a probabilistic sensitivity analysis (PSA) in the analysis [47], mainly because the parameters that are involved in the transmission dynamics are all correlated due to the calibration procedure, and these correlations are not known. ISPOR guidelines thus do not recommend a PSA for dynamic transmission modeling.

## 3. Results

### 3.1. Calibration Results

The model’s calibration results showed that the predicted IPD incidence since 2001 fit well with surveillance data upon visual inspection (see the Supplementary Material for the final calibrated parameters). We calculated the following coefficient of determination (CoD) of the incidence rate for each age group across serotype groups: $1-\sum_{i}{\left(y_{i}-\hat{y_{i}}\right)}^{2}/\sum_{i}{\left(y_{i}-\overset{-}{y}\right)}^{2}$, where $y_{i}$ is the surveillance value, $\hat{y_{i}}$ is the modeled value, and $\overset{-}{y}$ is the mean of the surveillance values. The CoDs for each age group were 0.954 (0–4 years), 0.777 (5–17 years), 0.835 (18–49 years), 0.806 (50–64 years), and 0.935 (≥65 years). Figures showing the model calibration stratified by age and serotype group may be seen in the Supplementary Material (Figure S1A–E).

### 3.2. Base-Case Results

The calibrated vaccine effectiveness against both carriage and IPD was highest for PCV7 serotypes. The model also estimated the vaccine effectiveness against IPD for serotypes 1, 5, 7F, and 6A as approximately 75% for each dose in the series. The estimated vaccine effectiveness against carriage for serotypes 1, 5, 7F, and 6A was 20.8%, 41.7%, and 65.0% for the first, second, and booster doses, respectively.

Table 4 presents the base-case model results. Over a 5-year period, with the current PCV13 1+1 vaccine program, the model estimated a cumulative total of 1,360,432 disease cases. With a PCV15 1+1 and PCV15 2+1 program, the model estimated total cases would decrease by 0.5% and 0.07% over the next five years, respectively. Comparatively, a PCV20 1+1 program was estimated to result in an 8.2% decrease in cases compared with a PCV13 1+1 program, whereas PCV20 2+1 program led to a 10.1% decrease in 5-year cumulative cases.

**Figure 2:**
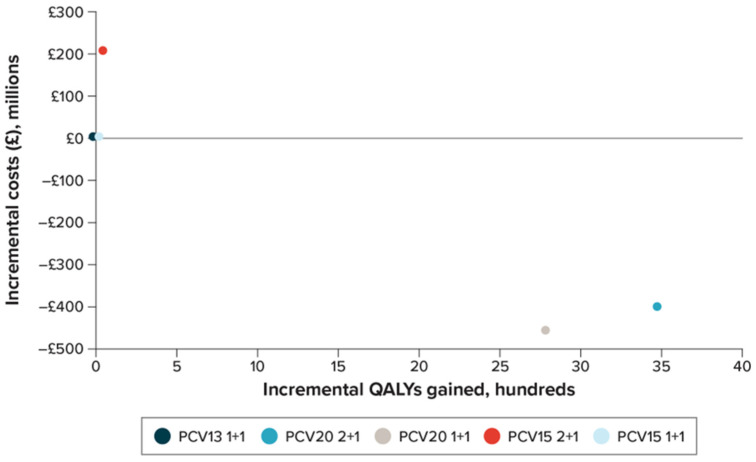
Cost-effectiveness plane. The incremental costs (in millions) versus incremental QALYs gained (in hundreds) for the evaluated programs. PCV13 1+1 was the baseline vaccine program, and the incremental results for all other vaccine programs are presented relative to PCV13 1+1.

Figure 2 presents the incremental cost-effectiveness plane for the evaluated schedules. Both PCV20 programs were found to be dominant (less costly and more effective) compared with the PCV15 and the PCV13 1+1 programs. PCV15 2+1 was more effective and more costly than PCV13 1+1 but was not cost-effective (incremental cost-effectiveness ratio [ICER] of over GBP 300,000 per QALY gained). PCV15 1+1 was cost-effective compared with PCV13 1+1 (at an ICER of GBP 3112 per QALY gained). PCV20 programs dominated PCV15 programs by being more effective and less costly than PCV15. Finally, compared with a PCV20 1+1 program, a PCV20 2+1 program was cost-effective, with an ICER of GBP 12,001/QALY gained (an NMB of GBP 82,199,578), which is well under the cost-effectiveness threshold range in the UK.

**Figure 3:**
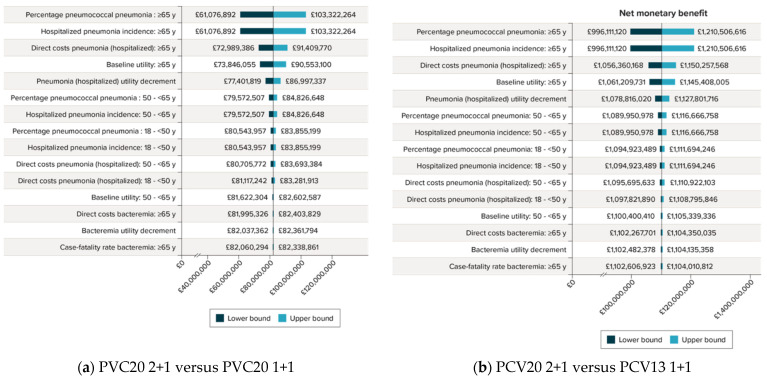
One-way sensitivity analyses on the net monetary benefit of PCV20 2+1 versus PCV20 1+1 and PCV13 1+1. The one-way deterministic sensitivity analysis was conducted by varying the standard error found in the data sources. When standard errors were not available, a ± 20%/1.96 was used to estimate the upper and lower bounds. Scenario Analysis Results.

One-way sensitivity analyses comparing PCV20 2+1 versus PCV20 1+1 and PCV13 1+1 are displayed in Figure 3. The top three most influential parameters in both comparisons were the percentage of pneumonia cases that are pneumococcal (individuals aged ≥65 years), the hospitalized pneumonia incidence (individuals aged ≥65 years), and the direct costs for hospitalized pneumonia (individuals aged ≥65 years). The NMB of PCV20 2+1 versus PCV20 1+1 was GBP 82,135,034, and the NMB did not fall below roughly GBP 61,000,000. For the comparison of PCV20 2+1 versus PCV13 1+1, the NMB was substantially more (about GBP 1 billion), and the NMB did not vary markedly from this estimate.

In scenario analysis, a PCV20 2+1 schedule was more effective than all other schedules in every scenario (Table 5). Compared with PCV15 1+1 and PCV13 1+1, PCV20 2+1 was dominant in all cases. PCV20 2+1 was cost-effective compared with PCV20 1+1 except when assuming equal booster dose effectiveness (i.e., booster dose effectiveness in a 1+1 program equaling the effectiveness in a 2+1 program). Including societal costs produced similar results to the base-case scenario.

## 4. Discussion

To our knowledge, this is the first published economic analysis of PCV15 and PCV20 in UK children. The results of the calibration showed that the historical trend leading up to the prospective results fit well with IPD surveillance using both visual inspection and a CoD. The prospective results showed that PCV20 2+1 was estimated to be dominant compared with PCV13, PCV15 1+1, and PCV15 2+1. Scenario analyses confirmed the dominance of PCV20 2+1. Additionally, PCV20 2+1 was estimated to be cost-effective compared with PCV20 1+1. In scenario analyses, the ICER of PCV20 2+1 versus PCV20 1+1 was slightly higher (though still under GBP 20,000/QALY) when assuming a 10% greater vaccine effectiveness for the additional covered serotypes in PCV20 over PCV13, given that it would provide greater marginal benefit to the less effective 1+1 program than to the 2+1 program.

This analysis found similar economic value (i.e., dominance) for PCV20 versus PCV13 as was observed in Public Health England’s original PCV13 model (PCV13 vs. PCV7) [12]. This is as expected, given each analysis examined the potential impact of implementing a novel vaccine providing coverage over previously vaccine-naive serotypes. Similar modeling efforts considering PCV13 dosing schedules in the UK did not generate as favorable results, as they explored scenarios in which the UK had already implemented PCV13 for several years, thus reducing the PCV13-specific disease burden [7]. As such, the comparison of the PCV20 2+1 schedule versus the 1+1 schedule yielded a more favorable economic result than the previous comparisons of PCV13 2+1 versus 1+1 [7,11].

Compared with PCV20, PCV15 did not present dominance versus PCV13. In comparison with PCV13, PCV15 provides additional protection against serotypes 22F and 33F, and the approximate IPD incidence of these serotypes is 0.8/100,000. As such, the impact on IPD of PCV15 relative to PCV13 is minimal, which makes offsetting increases in vaccine costs more difficult. PCV20 covers an additional five serotypes, of which serotype 8 represents a substantial proportion of disease. Thus, the incremental improvement in outcomes is much greater for PCV20.

Previous analyses focusing on the comparison between 2+1 and 1+1 schedules were conducted for PCV13 [7,11]. The assumption of equivalent booster dose protection for PCV13 1+1 is possibly less problematic, as nearly a decade of vaccination protection with a PCV13 2+1 schedule has been observed. For PCV15 and PCV20, the assumptions surrounding booster dose effectiveness become relevant, as the additional serotypes have never been previously covered. The calibration for this analysis did not include the reduction in IPD incidence seen during the first years of the COVID-19 pandemic. However, the reemergence of IPD incidence to levels seen before the pandemic makes the importance of modeling the COVID-19 pandemic less essential to the prospective analysis [10]. Additionally, because the switch to a 1+1 schedule occurred simultaneously with the COVID-19 pandemic, the implications of that switch on the reemergence of a PCV13-type disease are unclear. The rapid rebound of disease in the UK and persistent carriage in other countries suggest there is more complexity in carriage transmission and disease than previously considered in these models [9,10]. Finally, as PCV13’s real-world effectiveness in a 1+1 schedule is not yet validated due to its introduction coinciding with the onset of the COVID-19 pandemic and subsequent COVID-19 preventative measures, caution should be taken in forecasting disease between 1+1 and 2+1 schedules for a novel vaccine program. Additionally, IPD incidence is re-emerging faster than previously predicted [48].

The modeled analysis showed that PCV20 2+1 is cost-effective compared with a 1+1 schedule. The strength of this analysis relies on the use of the most recent publicly available data to fuel a DTM, which models both primary and downstream infections. A limitation of this analysis is the lack of published data on carriage prevalence. We were thus unable to calibrate to the carriage prevalence, and instead, we calibrated to IPD incidence. Moreover, there is also a lack of detailed contemporary published insight into individual serotype incidence distributions. Additionally, the IPD incidence rebound following the beginning of the COVID-19 pandemic raises questions about the estimates surrounding the duration of carriage. Indeed, data from other countries indicate carriage may have remained constant during the COVID-19 pandemic, leading to the postulate that our understanding of carriage dynamics may be flawed [49,50]. Another limitation in modeling the economic effects of pneumococcal vaccination is including nonhospitalized CAP data, as these data are extremely limited. Because of this, we excluded nonhospitalized CAP costs as a conservative assumption. There also exists limited data on real-world vaccine effectiveness, the duration of protection, and the waning of effectiveness. As such, we calibrated these parameters to best fit available IPD surveillance data using other known parameters from the literature. Finally, we assumed carriage of only one serotype at a time, which might underestimate carriage prevalence. However, a published report indicates stable co-colonization may be rare [51].

## 5. Conclusions

This analysis suggests that implementing a PCV20 schedule in the UK could substantially reduce the clinical and economic burden of pneumococcal disease. PCV20 is likely to be a cost-saving strategy compared with switching to PCV15 or continuing the use of PCV13. Additionally, a second primary dose for PCV20 (a 2+1 schedule) is cost-effective compared with one primary dose (a 1+1 schedule). Policymakers should consider the potential substantial reduction in disease cases with PCV20, which may more than offset vaccination costs. Because of the uncertainty in the dynamics surrounding carriage, the impact of the vaccine effectiveness against carriage in a 1+1 schedule, and the absence of any previous population-level protection against the novel serotypes in PCV20, implementing PCV20 in a 1+1 schedule (rather than a 2+1 schedule) should be considered with extra caution.

## Figures and Tables

**Table 1:** Model input parameters.

Parameter	Age Group, y				
	0–4	5–17	18–49	50–64	≥65
Population ^a^	1,342,117	9,874,836	26,085,921	12,601,911	10,297,259
All-cause mortality per 100,000 ^b^	201	15	94	500	4322
Overall IPD incidence per 100,000 ^c^	7.95	1.51	3.37	10.60	28.84
Hospitalized CAP incidence per 100,000 ^d^	355.07	43.25	121.62	439.95	3105.90
Mild otitis media incidence per 100,000 ^e^	5564.55	N/A	N/A	N/A	N/A
Moderate/severe otitis media incidence per 100,000 ^f^	243.50	N/A	N/A	N/A	N/A
Percentage of IPD that is meningitis ^g^	73.6%	57.7%	1.9%	1.9%	1.2%
Percentage of pneumonia pneumococcal ^h^	20%	20%	39.8%	39.8%	39.8%
Percentage of otitis media pneumococcal ^i^	20%	N/A	N/A	N/A	N/A
Case fatality rates ^j^					
IPD	0.148	0.063	0.123	0.159	0.304
Hospitalized pneumonia	0.002	0.016	0.049	0.140	0.367
Vaccine adherence (%) (1st primary dose/2nd primary dose/booster dose) ^k^					
2006–2010	95.0/95.0/85.0				
2010 onwards	97.0/97.0/91.0				
Vaccine costs, per dose (GBP)					
Administration	10.06				
PCV13	49.10				
PCV15	50.30				
PCV20	56.50				
Direct costs (GBP) ^l^					
Bacteremia	4214	4214	7010	7010	7010
Meningitis	6245	6245	8608	8608	8608
Otitis media (mild)	67	N/A	N/A	N/A	N/A
Otitis media (moderate/severe)	737	N/A	N/A	N/A	N/A
Pneumonia (hospitalized)	3756	3756	6472	6472	6472
Utility weights ^m^					
Baseline utility	0.9564	0.9564	0.9564	0.9335	0.8900
Disutility of bacteremia/meningitis	0.13	0.13	0.13	0.13	0.13
Disutility of hospitalized pneumonia	0.13	0.13	0.13	0.13	0.13
Disutility of otitis media	0.0035	0.0035	0.0035	0.0035	0.0035

CAP = community-acquired pneumonia; N/A = not applicable; PCV13 = 13-valent pneumococcal conjugate vaccine; PCV15 = 15-valent pneumococcal conjugate vaccine; PCV20 = 20-valent pneumococcal conjugate vaccine. ^a^ Office for National Statistics (ONS) [22]. ^b^ Office for National Statistics (ONS) [23]. ^c^ Ladhani et al. [8]. ^d^ Mohanty et al. [21] (for ages 0–17 years); and HES (for ages ≥18 years) [24]. ^e^ Mohanty et al. [25] (for ages 0–17 years); and The Health Improvement Network (THIN) database (for ages ≥18 years) [26]. ^f^ Mohanty et al. [25] (for ages 0–17 years); and THIN database (for ages ≥18 years) [26]. ^g^ Mohanty et al. [21] (for ages 0–17 years); and Miller et al. [27] (for ages ≥18 years). ^h^ For adults, percentages were obtained from Pick et al. [28]. For those under the age of 18 years, the percentage was assumed to be 20%. ^i^ Assumption. ^j^ Delgleize et al. [29]. ^k^ Adherence was based on NHS data [30]. For 1+1 programs, adherence to the 2nd priming dose was set to 0%. ^l^ The average cost per hospital admission for bacteremia, meningitis, and pneumonia was derived using average costs per finished consultant episode (FCE) from the national schedule of National Health Service (NHS) costs 2020/2021 [31] and the average number of FCEs per admission from NHS Digital, Hospital Episode Statistics for England, Admitted Patient Care statistics, 2020-21 [32]. Costs for otitis media were obtained from NHS reference costs 2021/22 [31]. ^m^ Baseline utility weights are from Ara and Brazier [33]. Disutility weights of IPD and pneumonia are from Mangen et al. [34]. Disutility weights of otitis media are from van Hoek et al. [12].

**Table 2:** Calibrated input parameters for vaccine effectiveness and duration of protection.

Parameter	1st Primary Dose	2nd Primary Dose	Booster Dose
Vaccine effectiveness against IPD (overall), % ^a^			
Serotype 8	74.3	74.3	75.0
Serotype 3	20.9	56.8	71.2
Serotypes 1, 5, 7F, 6A	74.3	74.3	75.0
Serotype 19A	69.4	98.0	98.6
PCV7 serotypes	81.7	95.5	98.8
Serotypes 22F and 33F	74.3	74.3	75.0
Serotypes 10A, 11A, 12F, and 15B	74.3	74.3	75.0
Vaccine effectiveness against carriage, % ^a^			
Serotype 8	20.8	41.7	65.0
Serotype 3	0.7	5.2	5.9
Serotypes 1, 5, 7F, 6A	20.8	41.7	65.0
Serotype 19A	2.7	20.8	21.2
PCV7 serotypes	35.0	77.8	88.8
Serotypes 22F and 33F	20.8	41.7	65.0
Serotypes 10A, 11A, 12F, and 15B	20.8	41.7	65.0
Mean duration of protection, years	0.7	0.7	1.4

IPD = invasive pneumococcal disease; PCV7 = 7-valent pneumococcal conjugate vaccine. ^a^ In the model, vaccine effectiveness to overall IPD ($VE_{OI}$) was calculated as $VE_{OI}=1-(1-VE_{IPD})(1-VE_{C})$, where $VE_{IPD}=$ the vaccine effectiveness against the IPD given carriage, and $VE_{C}=$ the vaccine effectiveness against carriage.

**Table 3:** Scenario Analyses in the Model.

Scenario Description	Modified Inputs
Varying the vaccine effectiveness against carriage and against IPD for the additional serotypes covered in PCV20 versus PCV13 by +/− 10%	Vaccine effectiveness to carriage and vaccine effectiveness to IPD
Assuming no difference in booster dose vaccine effectiveness against carriage between 2+1 and 1+1 schedules	No reduction in booster dose effectiveness
Inclusion of societal costs (see Supplementary Material)	Additional inputs of hourly wages and hours of lost productivity per case for persons/caregivers (see Table S5)

**Table 4:** Cost-effectiveness results (5-year time horizon).

Results	PCV13 1+1	PCV15 1+1	PCV15 2+1	PCV20 1+1	PCV20 2+1
No. of disease cases					
Bacteremia	8400	8385	8375	7599	7407
Meningitis	23,419	23,376	23,347	21,186	20,651
Otitis media	309,721	304,747	302,919	286,565	281,731
Pneumonia	1,018,891	1,017,705	1,016,844	933,585	913,092
Total cases	1,360,432	1,354,212	1,351,485	1,248,936	1,222,881
No. of deaths					
IPD	7348	7337	7330	6702	6547
Pneumonia	329,334	329,044	328,812	303,356	297,104
Outcomes					
Life-years	292,019,917	292,020,179	292,020,392	292,043,082	292,048,735
QALYs	249,069,025	249,069,386	249,069,665	249,097,121	249,104,034
Costs					
Vaccination	GBP 389,480,098	GBP 397,380,303	GBP 601,882,252	GBP 427,783,464	GBP 605,082,671
IPD	GBP 199,208,904	GBP 198,880,014	GBP 198,654,045	GBP 180,839,611	GBP 176,372,013
Pneumonia	GBP 5,910,059,997	GBP 5,904,041,761	GBP 5,899,346,527	GBP 5,432,747,000	GBP 5,316,351,443
Otitis media	GBP 25,105,771	GBP 24,677,615	GBP 24,526,927	GBP 23,292,008	GBP 22,921,733
Total	GBP 6,523,854,771	GBP 6,524,979,693	GBP 6,724,409,752	GBP 6,064,662,083	GBP 6,120,727,860
ICER					
vs. PCV13		GBP 3112	GBP 313,229	Dominant	Dominant
vs. PCV15 1+1			GBP 715,179	Dominant	Dominant
vs. PCV15 2+1				Dominant	Dominant
vs. PCV20 1+1					GBP 8110

ICER = incremental cost-effectiveness ratio; QALY = quality-adjusted life-year.

**Table 5:** Scenario analysis results comparing total costs and QALYs of PCV20 2+1 with PCV15 and PCV13.

Scenario	Outcome	PCV20 2+1	PCV13 1+1	PCV15 1+1	PCV20 1+1	PCV15 2+1
Base case	Total cost	GBP 6,116,860,165	GBP 6,523,854,771	GBP 6,517,281,841	GBP 6,060,729,843	GBP 6,717,270,837
	Total QALYs	249,104,034	249,069,025	249,069,477	249,097,121	249,069,665
	ICER		Dominant	Dominant	GBP 8119	Dominant
PCV20-13 VE 10% higher	Total cost	GBP 6,096,801,176	GBP 6,523,854,771	GBP 6,524,319,132	GBP 6,037,091,426	GBP 6,723,498,496
	Total QALYs	249,105,398	249,069,025	249,069,421	249,098,684	249,069,715
	ICER		Dominant	Dominant	GBP 8893	Dominant
PCV20-13 VE 10% lower	Total cost	GBP 6,148,586,783	GBP 6,523,854,771	GBP 6,525,643,282	GBP 6,095,526,080	GBP 6,725,327,097
	Total QALYs	249,102,447	249,069,025	249,069,351	249,095,372	249,069,615
	ICER		Dominant	Dominant	GBP 7500	Dominant
Equal booster VEc	Total cost	GBP 6,116,860,165	GBP 6,523,854,771	GBP 6,523,427,466	GBP 5,993,157,158	GBP 6,717,270,837
	Total QALYs	249,104,034	249,069,025	249,069,477	249,101,199	249,069,665
	ICER		Dominant	Dominant	GBP 44,994	Dominant
Societal perspective	Total cost	GBP 6,370,442,692	GBP 6,806,691,873	GBP 6,806,783,657	GBP 6,320,777,968	GBP 7,005,697,126
	Total QALYs	249,104,034	249,069,025	249,069,386	249,097,121	249,069,665
	ICER		Dominant	Dominant	GBP 7184	Dominant

VE = vaccine effectiveness; VEc = vaccine effectiveness against carriage.

## Data Availability

All data generated or analyzed during this study are included in this published article/as supplementary information files.

## Supplementary Materials

The following supporting information can be downloaded at: https://www.mdpi.com/article/10.3390/vaccines11071168/s1, Figure S1: Invasive pneumococcal disease incidence per 100,000 patients by serotype group from 2001 to 2019: calibrated versus surveillance; Table S1: Calibrated input parameters for the duration of carriage and rate of developing IPD given carriage (per 100,000 acquisitions); Table S2: Calibrated input parameters for the probability of carriage given contact with a carrier absent vaccination; Table S3: Contact matrix; Table S4: Detailed epidemiological results (5-year time horizon); Table S5: Indirect costs [20,29,52,53].
