# Supplementary material for: Estimating the Cost-Effectiveness of Switching to Higher-Valency Pediatric Pneumococcal Conjugate Vaccines in the United Kingdom
Source: Vaccines (Basel). 2023 Jun 28;11(7):1168. doi: 10.3390/vaccines11071168 (PMC10386052; doi:10.3390/vaccines11071168)
Supplement: Supplementary file 1 [file vaccines-11-01168-s001.zip › vaccines-2439974-supplementary.pdf]

Supplementary Material

CALIBRATION PROCEDURE

We assumed that invasive pneumococcal disease (IPD) incidence was in a steady state before the introduction of the 7-valent pneumococcal conjugate vaccine (PCV7). We therefore used a steady-state approximation to calculate the probability of acquiring a serotype given contact with a carrier absent vaccination for each serotype and selected age group. A steady-state approximation allows the model to estimate rates so that the annual number of carriage acquisitions equals the annual number of individuals who clear the serotype, with no births or deaths.

Using these rates, the model uses a simulated annealing approach to draw calibrated parameters within certain bounds, run the model forward from 2001, and calculate the IPD incidence curves by age and serotype group. Briefly, simulated annealing allows the model to draw a subsequent calibrated parameter distribution that is “not too far” from the most recent parameter draw that gave “good” results (i.e., one that has a low value for the objective function: the difference of the sum of squared deviations of the resulting yearly IPD incidence values produced from the model with the actual IPD surveillance values). The model chooses the final set of calibrated parameters as the one that minimizes the objective function over a set of approximately 1,000 draws. The final set of calibrated parameters, along with initial distribution of the population in the model compartments given all parameters and model dynamics, are used to run the model “forward” (i.e., projecting into the future).

COSTS FOR HOSPITAL ADMISSIONS DUE TO IPD AND CAP

The average costs per hospital admission for bacteriemia, meningitis, and pneumonia were derived using average costs per finished consultant episode (FCE) from the national schedule of National Health Service (NHS) costs 2020/2021, and the average number of FCEs per admission

from NHS Digital, Hospital Episode Statistics for England, Admitted Patient Care statistics, 2020-21.

The activity-weighted national average cost per FCE and the average number of FCEs per admission were estimated for each manifestation using the relevant Healthcare Resource Group (HRG) codes. For children, the number of FCE's per admission was assumed to equal 1 given the estimated median length of stay in hospital of 1 day in the British Thoracic Society 2016/2017 audit [1].

- For bacteremia, *International Classification of Diseases, Tenth Revision* (ICD-10) codes A40.3, A40.8, A40.9, A41.9, A49.1, A49.9, and I33.0 were mapped to HRG codes WJ06, WJ03, WJ02, and EB02 for adult patients and PW16, PW17, and PE23 for pediatric patients.
- For meningitis, ICD-10 codes G00.1, G00.8, G00.9, G04.2, G04.8, G04.9, and G05.0 were mapped to HRG code AA22 for adult patients and PW16 for pediatric patients.
- For pneumonia, ICD-10 codes J13, J15.9, and J18 were mapped to HRG codes DZ11 and DZ23 for adult patients and PD14 for pediatric patients.

Table S1. Calibrated input parameters for the duration of carriage and rate of developing IPD given carriage (per 100,000 acquisitions).

	Age group, y					
**Parameter**	**0 - <2**	**2-4**	**5-17**	**18-49**	**50-64**	**≥65**
Mean duration of carriage, wk						
Serotype 8	5.3	4.4	3.6	1.7	2.0	5.3
Serotype 3	3.2	3.3	1.2	3.2	4.9	3.2
Serotypes 1, 5, 7F, 6A	2.3	1.8	2.4	2.6	4.1	2.3
Serotype 19A	2.1	6.2	5.6	1.8	2.5	2.1
PCV7-serotypes	7.3	2.2	5.3	7.2	6.4	7.3
Serotypes 22F and 33F	3.1	4.0	2.5	5.3	5.9	3.1
Serotypes 10A,11A,12F, and 15B	2.5	2.8	2.0	8.9	4.7	2.5
Non-vaccine serotypes	2.5	2.4	4.5	1.8	1.8	2.5
Rate of developing IPD given carriage (per 100,000 acquisitions)						
Serotype 8	1.3	73.5	89.5	140.7	82.2	1.3
Serotype 3	18.2	36.8	85.5	19.2	64.0	18.2
Serotypes 1, 5, 7F, 6A	44.7	68.1	48.6	66.2	76.1	44.7
Serotype 19A	5.0	80.1	16.7	148.5	23.4	5.0
PCV7-serotypes	102.3	41.0	127.7	154.2	75.8	102.3
Serotypes 22F and 33F	86.0	35.2	152.8	15.4	52.1	86.0
Serotypes 10A,11A,12F, and 15B	114.0	125.3	92.3	109.7	80.4	114.0
Non-vaccine serotypes	199.1	5.9	7.3	35.1	7.8	199.1

PCV7 = 7-valent pneumococcal conjugate vaccine; IPD = invasive pneumococcal disease.

Table S2. Calibrated input parameters for the probability of carriage given contact with a carrier absent vaccination.

Parameter, %	Age group, y						
	**<1**	**1-2**	**2-4**	**5-17**	**18-49**	**50-64**	**≥65**
Serotype 8	1.78	2.14	1.90	0.03	0.16	0.36	3.44
Serotype 3	0.20	0.24	0.21	0.02	0.04	0.80	0.98
Serotypes 1, 5, 7F, 6A	1.33	1.59	1.41	0.52	1.52	1.58	2.44
Serotype 19A	3.38	4.05	3.59	0.08	0.48	0.25	4.56
PCV7-serotypes	0.81	0.97	0.86	0.29	0.23	0.47	2.27
Serotypes 22F and 33F	0.29	0.35	0.31	0.09	0.09	1.93	2.22
Serotypes 10A,11A,12F, and 15B	0.77	0.93	0.82	0.10	0.49	0.84	2.98
Non-vaccine serotypes	0.05	0.06	0.05	0.29	0.76	0.64	8.32

PCV7 = 7-valent pneumococcal conjugate vaccine.

Table S3. Contact matrix [a].

	Age group									
**Age group**	**0-1 mo**	**2-3 mo**	**4-5 mo**	**6-11 mo**	**1-2 y**	**2-4 y**	**5-17 y**	**18-49 y**	**50-64 y**	**≥65 y**
0-1 mo	18.13	18.13	18.13	18.13	18.13	18.13	4.44	5.31	2.80	2.01
2-3 mo	18.13	18.13	18.13	18.13	18.13	18.13	4.44	5.31	2.80	2.01
4-5 mo	18.13	18.13	18.13	18.13	18.13	18.13	4.44	5.31	2.80	2.01
6-11 mo	54.39	54.39	54.39	54.39	54.39	54.39	13.31	15.92	8.40	6.02
1-2 y	90.64	90.64	90.64	90.64	90.64	90.64	22.18	26.54	13.99	10.04
2-4 y	326.31	326.31	326.31	326.31	326.31	326.31	79.85	95.54	50.37	36.14
5-17 y	458.44	458.44	458.44	458.44	458.44	458.44	1,483.58	459.56	197.59	214.99
18-49 y	910.31	910.31	910.31	910.31	910.31	910.31	603.93	714.72	576.21	323.39
50-64 y	255.50	255.50	255.50	255.50	255.50	255.50	146.28	182.39	302.95	197.10

[a] The average number of contacts per year for each age category in the epidemiological model, derived from Mossong et al. [2].

Table S4. Detailed epidemiological results (5-year time horizon).

Result	PCV13 1+1	PCV15 1+1	PCV15 2+1	PCV20 1+1	PCV20 2+1
Disease cases (<5 y old), n					
Bacteremia	361	353	350	335	330
Meningitis	1,006	983	976	933	920
Otitis media	211,418	206,552	205,125	196,036	193,356
Pneumonia	12,925	12,627	12,540	11,984	11,821
Total cases	225,710	220,515	218,992	209,288	206,427
Disease cases (≥5 y old), n					
Bacteremia	8,039	8,032	8,024	7,265	7,077
Meningitis	22,413	22,393	22,371	20,253	19,731
Otitis media	98,303	98,195	97,794	90,530	88,375
Pneumonia	1,005,966	1,005,077	1,004,304	921,600	901,271
Total cases	1,134,722	1,133,697	1,132,493	1,039,648	1,016,454
Deaths (<5 y old), n					
IPD	202	198	196	188	185
Pneumonia	26	25	25	24	24
Deaths (≥5 y old), n					
IPD	7,348	7,337	7,330	6,702	6,547
Pneumonia	329,334	329,044	328,812	303,356	297,104

IPD = invasive pneumococcal disease; PCV13 = 13-valent pneumococcal conjugate vaccine; PCV15 = 15-valent pneumococcal conjugate vaccine; PCV20 = 20-valent pneumococcal conjugate vaccine.

Table S5. Indirect costs.

Parameter	Age group, y				
	0-4	5-17	18-49	50-64	≥65
Hours of lost productivity per case for persons/caregivers					
Bacteremia[a]	57.25	57.25	57.25	58.64	8.73
Meningitis[a]	97.39	97.39	97.39	99.74	14.85
Otitis media (mild)[b]	8.00	8.00	N/A	N/A	N/A
Otitis media (moderate/severe)[b]	8.00	8.00	N/A	N/A	N/A
Pneumonia (hospitalized)[a]	55.11	55.11	55.11	56.44	8.40
Average hourly wage[c]			£15.75		

N/A = not applicable.

[a] Delgleize et al. [3]; hours missed based on duration of illness multiplied by proportion of age group (or parental ages) working.

[b] Office for National Statistics [4]. Assumption of 1 day for resolving infection with 8 hours/day lost.

[c] Office for National Statistics [4]. Assumption of 40 hour work week.

A. Ages 0 to < 4 years

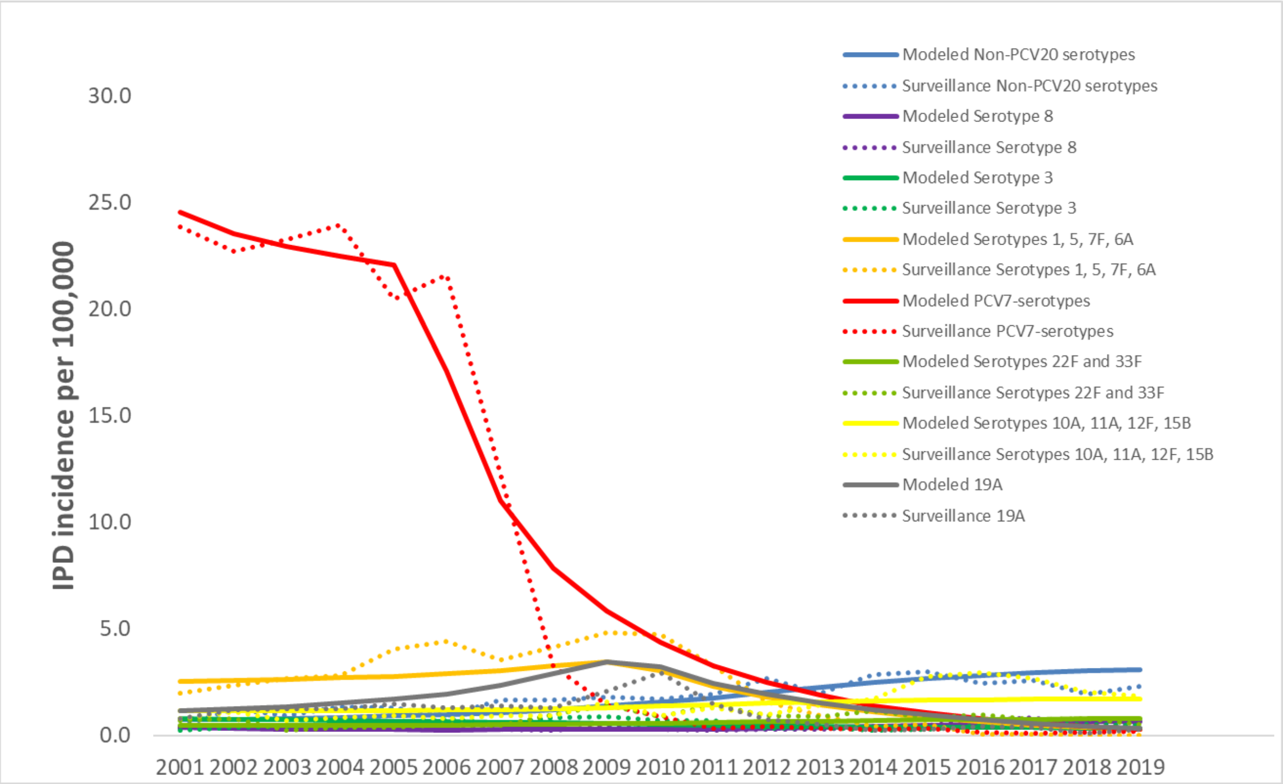

B. Ages 5 to 17 years

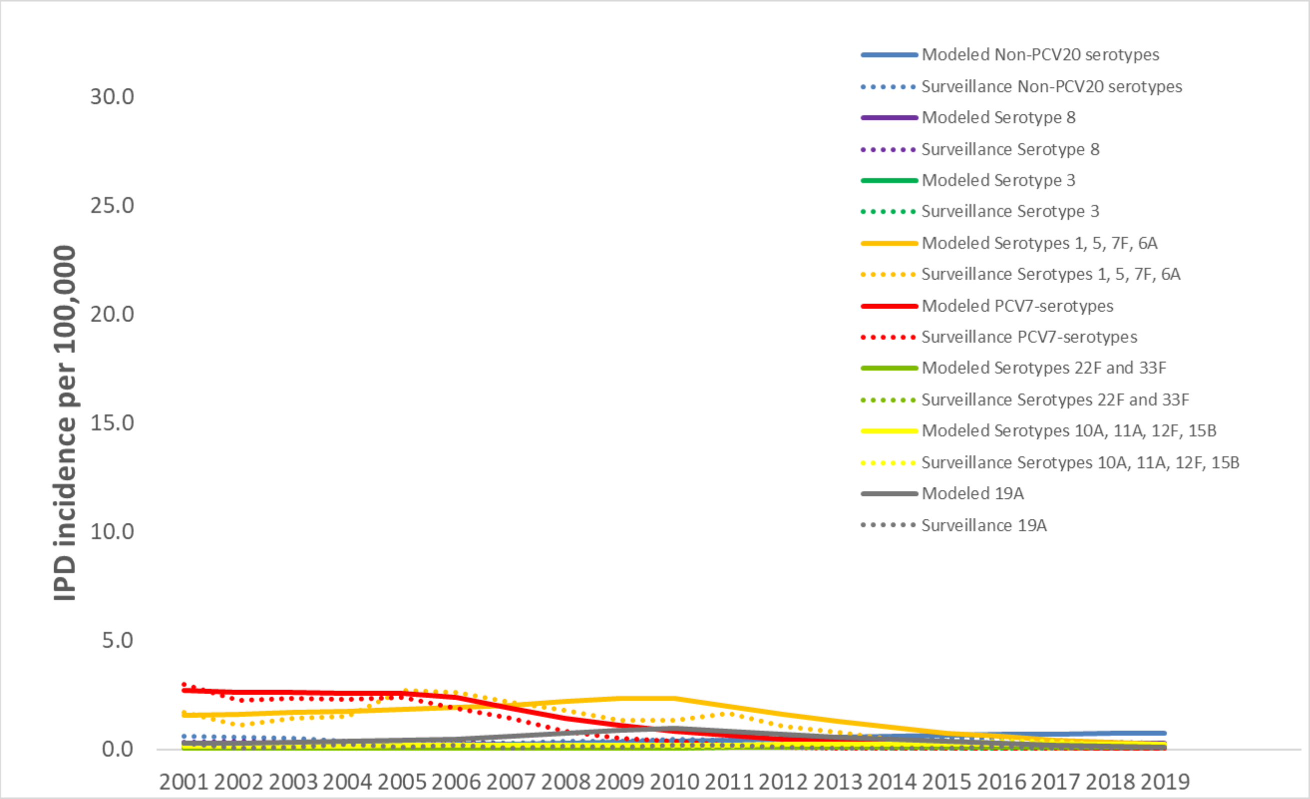

C. Ages 18 to 49 years

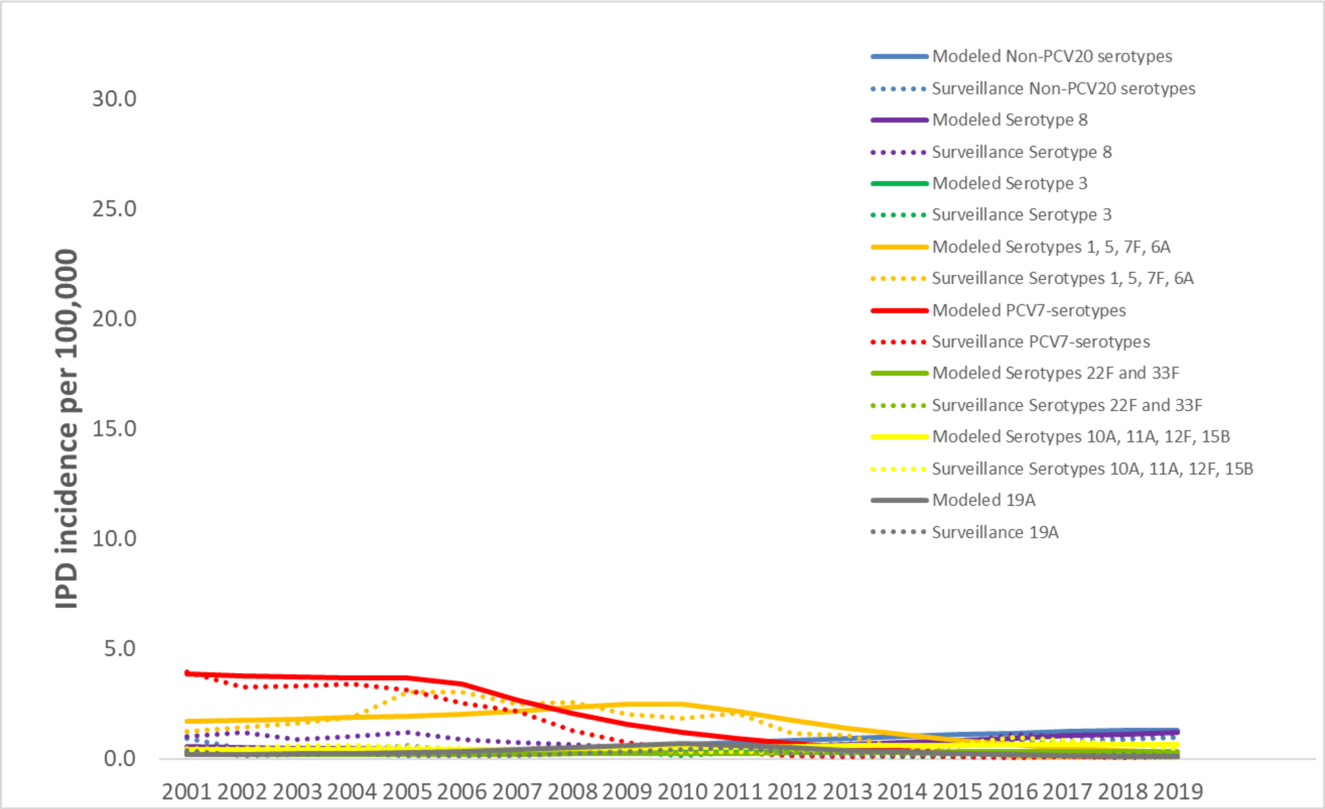

D. Ages 50 to 64 years

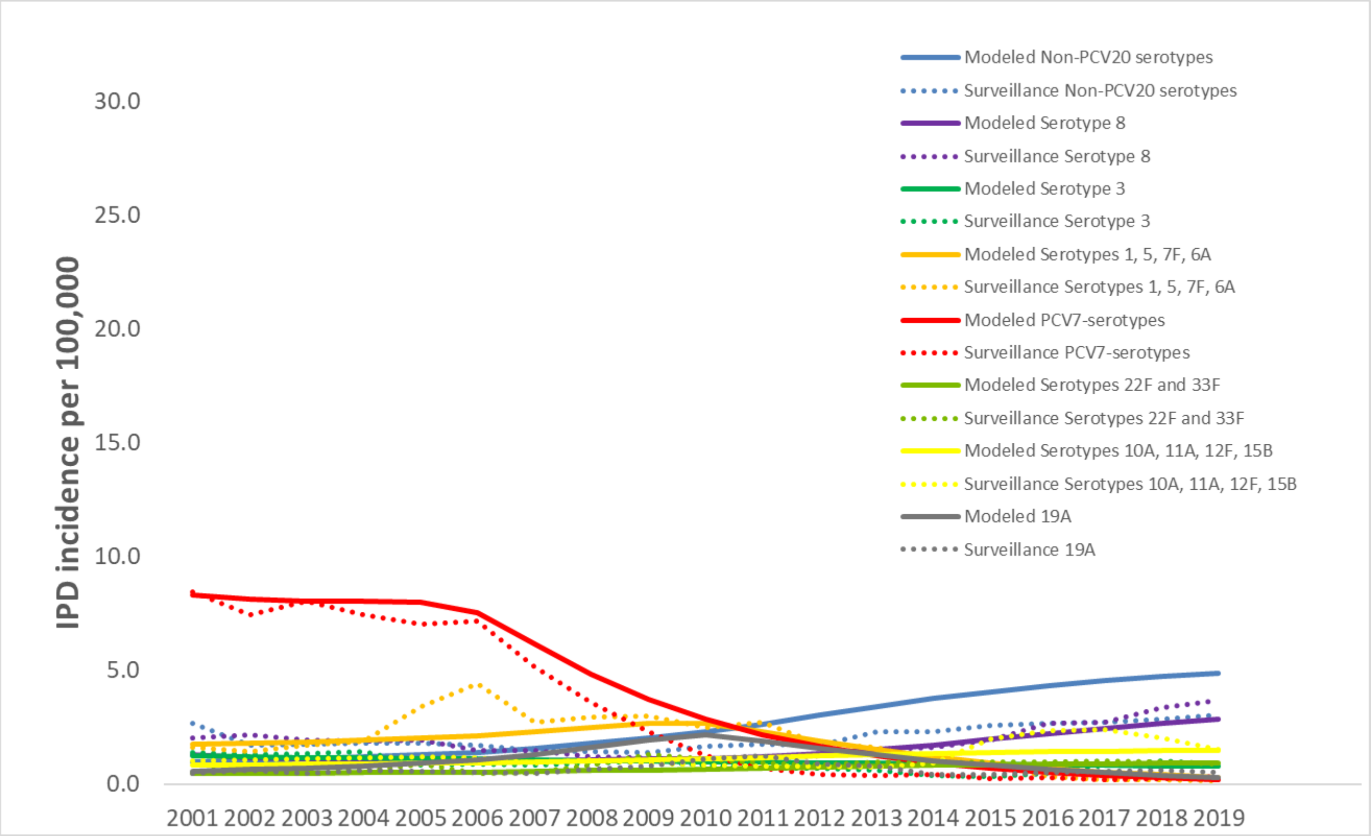

E. Ages ≥65 years

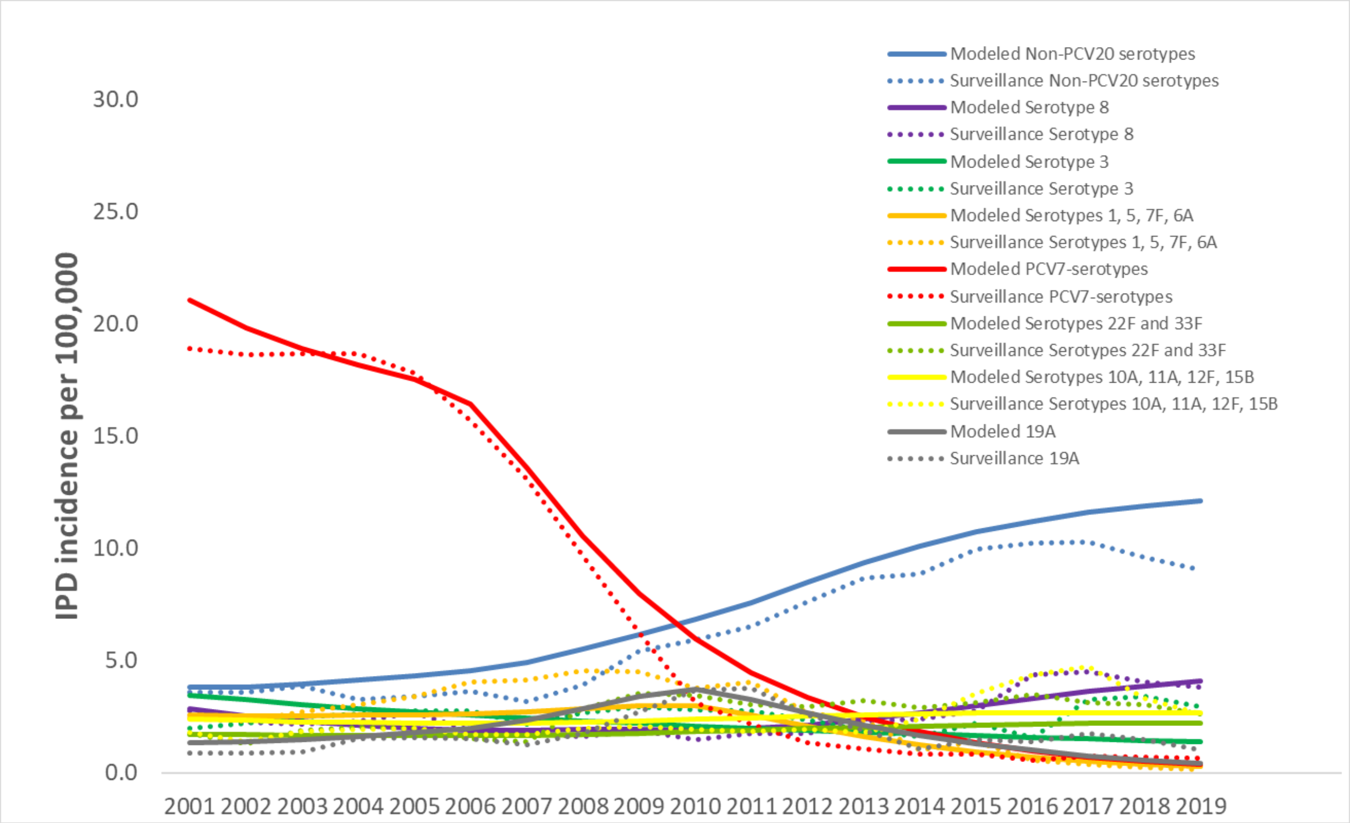

Figure S1. Invasive pneumococcal disease incidence per 100,000 patients by serotype group from 2001 to 2019: calibrated versus surveillance.

References

1. Legg J, Rampton C. British Thoracic Society. Paediatric pneumonia audit. National audit period: 1 November 2016 – 31 January 2017. 15 January 2018. https://www.brit-thoracic.org.uk/document-library/quality-improvement/audit-reports/paediatric-pneumonia-201617/. Accessed 2 March 2023.

2. Mossong J, Hens N, Jit M, Beutels P, Auranen K, Mikolajczyk R, et al. Social contacts and mixing patterns relevant to the spread of infectious diseases. PLoS Med. 2008 Mar 25;5(3):e74. http://dx.doi.org/10.1371/journal.pmed.0050074.

3. Delgleize E, Leeuwenkamp O, Theodorou E, Van de Velde N. Cost-effectiveness analysis of routine pneumococcal vaccination in the UK: a comparison of the PHiD-CV vaccine and the PCV-13 vaccine using a Markov model. BMJ Open. 2016 Nov 30;6(11):e010776. http://dx.doi.org/10.1136/bmjopen-2015-010776.

4. Office for National Statistics (ONS). EARN01: Average weekly earnings [dataset]. 2023. https://www.ons.gov.uk/employmentandlabourmarket/peopleinwork/earningsandworkinghours/datasets/averageweeklyearningsearn01. Accessed 2 May 2023.
